# The effects of common matrices for assay standards on performance of ‘ultra sensitive’ immunometric assays for TSH: Report of a joint WHO/IFCC collaborative study

**DOI:** 10.1155/S1463924691000354

**Published:** 1991

**Authors:** R. E. Gaines-Das, A. F. Bristow, H. Brettschneider

**Affiliations:** 1 WHO International Laboratory for Biological Standards National Institute for Biological Standards and Control Blanche Lane South Mimms, Hertfordshire Potters Bar EN6 3QG UK; 2 Boehringer Mannheim GmbH Werk Penzberg Nonnenwald 2 Penzberg/Obb D81122 Germany

## Abstract

This report describes the results of a collaborative study organized by a joint working group of the IFCC and WHO and involving nine manufacturers of TSH immunometric assay kits. The study was designed to determine whether a calibrator with a common matrix gives better between-laboratory agreement for calibration of serum samples than the various kit calibrators, and to assess various materials for their suitability for use as common matrices. Kit calibrators, or calibrators consisting of the IRP for TSH made up in two common matrices: (a) serum from patients with untreated thyrotoxicosis or (b) serum taken from subjects treated with suppressive doses of triiodothyronine, gave similar results for the between-laboratory variation of estimates of TSH concentration for a range of serum samples. Dose-response curves for the two calibrators in ‘common’ matrices were similar to one another and to those for the kit calibrator. However, the occurrence of non-specific serum effects is shown by the comparison of results for these calibrators with results for calibrators made up in a third common matrix: serum treated with wheat germ lectin. Dose response curves for this calibrator were dissimilar to those for the other calibrators and between-laboratory variation for estimates in terms of this latter calibrator showed a substantial increase. Moreover, although the between-laboratory variances for estimates of the TSH concentration in terms of each of these calibrators (except those made up in serum treated with the wheat germ lectin) were similar for any one sample from five hyperthyroid patients, the variances were not consistent between samples, even for samples with similar mean TSH concentrations. These results suggest that a major factor in the between-laboratory variation, especially in the region near ‘zero dose’, is sample-related, and is caused by particular samples interacting differently with different assay systems.

In general, it would appear that for the well-controlled ‘ultrasensitive’ TSH immunometric assay kits, included in this study, between-laboratory agreement of estimates of the TSH concentration in serum samples is not likely to be substantially improved by use of a common matrix for the standards.


Journal of Automatic Chemistry, Vol. 13, No. 5 (September–October 1991), pp. 209–215

### The effects of common matrices for assay standards on performance of 'ultra sensitive' immunometric assays for TSH: Report of a joint WHO/IFCC collaborative study

**R. E. Gaines-Das, A. F. Bristow**

*WHO International Laboratory for Biological Standards, National Institute for Biological Standards and Control, Blanche Lane, South Mimms, Potters Bar, Hertfordshire EN6 3QG, UK*

**and H. Brettschneider**

*Boehringer Mannheim GmbH, Werk Penzberg, Nonnenwald 2, D81122 Penzberg/Obb, Germany*

*This report describes the results of a collaborative study organized by a joint working group of the IFCC and WHO and involving nine manufacturers of TSH immunometric assay kits. The study was designed to determine whether a calibrator with a common matrix gives better between-laboratory agreement for calibration of serum samples than the various kit calibrators, and to assess various materials for their suitability for use as common matrices. Kit calibrators, or calibrators consisting of the IRP for TSH made up in two common matrices: (a) serum from patients with untreated thyrotoxicosis or (b) serum taken from subjects treated with suppressive doses of triiodothyronine, gave similar results for the between-laboratory variation of estimates of TSH concentration for a range of serum samples. Dose-response curves for the two calibrators in 'common' matrices were similar to one another and to those for the kit calibrator. However, the occurrence of non-specific serum effects is shown by the comparison of results for these calibrators with results for calibrators made up in a third common matrix: serum treated with wheat germ lectin. Dose response curves for this calibrator were dissimilar to those for the other calibrators and between-laboratory variation for estimates in terms of this latter calibrator showed a substantial increase. Moreover, although the between-laboratory variances for estimates of the TSH concentration in terms of each of these calibrators (except those made up in serum treated with the wheat germ lectin) were similar for any one sample from five hyperthyroid patients, the variances were not consistent between samples, even for samples with similar mean TSH concentrations. These results suggest that a major factor in the between-laboratory variation, especially in the region near 'zero dose', is sample-related, and is caused by particular samples interacting differently with different assay systems.*

*In general, it would appear that for the well-controlled 'ultra-sensitive' TSH immunometric assay kits, included in this study, between-laboratory agreement of estimates of the TSH concentration in serum samples is not likely to be substantially improved by use of a common matrix for the standards.*

### Abbreviations

TSH : Thyroid stimulating hormone (thyrotropin)
IRP : International reference preparation
$T_3$ : Triiodothyronine
IgG : Immunoglobulin-G
WGL : Wheat germ lectin
C.V. : Coefficient of variation

### Introduction

At the 36th meeting of WHO's Expert Committee on Biological Standardization, the need for matrix standards to improve calibration of ligand assay kits for estimation of hormones was considered (WHO, 1987), and it was agreed that a joint working group of IFCC and WHO be set up to conduct a feasibility study into this matter. This working group agreed that a collaborative study should be undertaken to investigate whether calibration of serum samples, in terms of the IRP for TSH in a common matrix, shows better between-laboratory agreement than calibration in terms of kit standards. The group discussed various materials which may be suitable for use as a common matrix and samples were obtained and examined in a collaborative study.

### Aims of the study

The aim of the study was to assses four low TSH serum preparations for their suitability for serving as a common matrix for TSH assays: (*a*) from untreated thyrotoxic patients; (*b*) from triiodothyronine-suppressed volunteers, in both frozen and lyophilized form; and (*c*) from samples where the glycoproteins had been removed by wheat germ lectin chromatography.

### Materials for the study

*(1) Matrices*

*Thyrotoxic serum:* Some 200 serum samples, ranging in volume from 2 to 40 ml, were obtained from untreated patients with long-term thyrotoxicosis, each containing less than 0·05 mU-L TSH by immunoassay, and no detectable anti-mouse IgG.

*$T_3$-suppressed serum:* Some 200 to 250 ml of serum was collected from each of 22 healthy volunteers who had taken suppresssive doses of triiodothyronine (80 μg per day) for five days prior to serum donation, each sample containing less than 0·05 mU/L TSH and no detectable anti-mouse IgG.

© WHO/IFCC 1991

*WGL serum:* Serum from healthy volunteers was mixed gently for 16 h with WGL-Sepharose-6MB (Pharmacia) and collected by filtration, lyophilized and reconstituted in water.

*(2) TSH standards*

The ampouled preparation 81/565 contains highly-purified pituitary TSH identical to that used in the 2nd International Reference Preparation (IRP), and is calibrated in terms of the 2nd IRP [1].

*(3) Patient samples*

Samples ranging from 25 to 50 ml were collected from each of 11 patients who were either hypothyroid, euthyroid or hyperthyroid, and these were filtered and distributed into 0·5 ml portions.

### Study design

The study was designed to calibrate a panel of clinical serum samples using both in-house TSH assay standards, i.e. kit calibrators, or 'calibrator sets' prepared in a common matrix, and to compare the intra- and inter-laboratory variability obtained by these calibrations. Each assay that individual participants performed contained the following standard curves:

(1) Individual kit-calibrator or in-house standard curve.

(2 and 3) Standard curves in duplicate, prepared in thyrotoxic serum by the organizers and distributed frozen to participants.

(4 and 5) Standard curves in duplicate, prepared in $T_3$-suppressed serum by the organizers and distributed frozen to participants.

(6) Standard curve, prepared in WGL serum by the organizers and distributed frozen to participants.

(7) Standard curve prepared in-house by each participant using $T_3$-suppressed serum as diluent, distributed frozen to participants.

(8) Standard curve prepared in-house by participants using freeze-dried $T_3$-suppressed serum as diluent, supplied by the organizers.

(9) Standard curve prepared in-house by participants using freeze-dried WGL serum as diluent, supplied by the organizers.

The samples comprising curves (2)–(6) were provided coded and in random order amongst the distributed serum samples and were not distinguishable from the 'patient' serum samples. In each assay, the TSH content of the unknowns was estimated using each of the standard curves.

Comparisons of the results obtained with curve (1) and curves (2)–(9) would indicate any improvement in comparability obtained by using a common standard curve prepared in a common matrix. Comparisons of results obtained from curves (2)–(6) with results from (7)–(9) would allow separation of that improvement into components derived from the use of a common matix, and from the removal of in-house dilution errors. Assessment of results obtained from curves (7), (8) and (9) would indicate whether more widely available TSH-free matrices in either freeze-dried or frozen form would be suitable for use as common assay matices.

### Participants

Nine laboratories participated in the study, and for convenience in analysis have been referred to by a code number from 1 to 9, which does not relate to the order of listing below. Each of the participants was a manufacturer of immunometric TSH immunoassay kits. Participants contributing data were:

Dr A. Swift, Diagnostic Products, 31 Station Lane, Witney, Oxon OX8 6AN, UK.
Dr R. Palmer, Serono Diagnostics Ltd, Woking Business Park, Woking, Surrey GU21 5JY, UK.
Dr S. R. Abbott, Boots-Celltech Diagnostics Ltd, 240 Bath Road, Slough, Berkshire SL1 4ET, UK.
Dr Gard Schnorr, Hoechst AG, Radiochemisches Laboratorium, Postfach 80 03 20, D6230 Frankfurt/Main 80, Germany.
Dr A. Massaglia, Diagnostics Division, Sorin Biomedica SpA, Str. per Crescentino 13040, Saluggia (VC), Italy.
Dr H. Brettschneider, Boehringer Mannheim GmbH, Abt. D-DQ, D8122 Penzberg, Nonnenwald, Germany.
Drs Rudolf Nothelfer and Schuster, Henning Berlin GmbH, Abt. Diagnostika, Komturstr. 58–62, D1000 Berlin 42, Germany.
Dr K. Pettersson, Wallac-Pharmacia P.O. Box 10, SF-20101 Turku 10, Finland.
Mr J. Harris, Amerlite Quality Control, Amersham International plc, Forest Farm, Whitchurch, Cardiff CF4 7YT, UK.

*Samples provided to participants*

A summary of the sample together with their description and the 'code name' used for analysis and throughout this report is given in table 1.

*Assays requested*

Each participant was requested to carry out three assays, each including all samples listed above with at least duplicate determinations for each sample.

Participants were also requested to include their in-house calibrators made up in the routine manner, code named 'KIT' for the analysis.

*Reporting of data*

All raw data, preferably as counts, extinction values, etc., were returned on centrally distributed data forms together with details of the assays.

*Table 1. Coding of samples provided to participants.*

| Code used for analysis and report | Description of sample | Comments |
|---|---|---|
| *Centrally prepared calibrator sets* | | |
| TTA | Calibrator sets independently | Each calibrator set |
| TTB | prepared in thyrotoxic serum | contained TSH |
| T3A | Calibrator sets independently | concentrations of |
| T3B | prepared in T3-suppressed serum | 0, 0·11, 0·34, 1·03 |
| WGA | Calibrator set prepared in wheat-germ treated serum | 3·08, 10·3, 30·8 mU/L |
| *Calibrator sets prepared in-house* | | |
| T3F | Calibrator set prepared in frozen T3-suppressed serum | Each calibrator set |
| T3Y | Calibrator set prepared from lyophilized T3-suppressed serum | contained TSH concentrations of |
| WGX | Calibrator set prepared from lyophilized wheat-germ lectin-treated serum | 0, 0·11, 0·33, 1, 3, 10 and 30 mU/L |
| KIT | KIT calibrators | |
| *Samples* | | |
| ER1–5 | Hyperthyroid samples | |
| N1–4 | Normal (Euthyroid) samples | |
| N1D, N3D | Duplicates of N1 and N3 | |
| O1, O2 | Hypothyroid samples | |

Centrally prepared calibrator sets and samples were provided as 0·5 ml frozen aliquots with the only identification being a number between 1 and 55. The matrices for in-house calibrator sets (T3F, T3Y and WGX) were identified to participants as T3-suppressed serum, Y and X respectively. Preparation of in-house calibration was according to a provided protocol. Assay layout was randomised according to a centrally-designed format. Both centrally-prepared and in-house calibrator sets were prepared using the ampoules TSH preparation 81/565 [1].

*Methods of statistical analysis*

The analysis consisted of several stages. Initially, the reported raw responses were examined for response error relationships and for any notable outliers both graphically using plots of variance of responses against mean responses, and statistically, using the program SCAN [2]. A maximum likelihood method was used for fitting the parameter k of a response error relationship of the form variance of y proportional to $y^k$ [3]. Also at this stage raw responses for the duplicated frozen samples (code names N1 and N1D, T3P and T3PD, N3 and N3D) were compared using analysis of variance within results for each laboratory to detect any significant monotonic drift in response data, and raw responses for the 'blanks' with presumed nil concentraion of TSH, namely the 'zero dose' concentrations for each calibrator set and the hyperthroid patient samples, were analysed and compared.

Secondly, common asymptotes based on four parameter logistic curves were determined for each assay and used to transform the raw responses to logits. Weighted linear regresssion was then used to fit log dose-logit response lines for each calibrator (code names KIT, TTA, TTB, T3A, T3B, WGA, T3F, WGX and T3Y) [4]. The resulting lines were examined, both graphically and statistically, for linearity and parallelism within assays. The within-assay ranks of the slopes of these lines were compared across assays within laboratories for any consistency of ordering.

The 'potencies' of these various calibrators relative to one another were calculated using the WRANL programme for the analysis of parallel line assays [5], and compared within and between laboratories using analysis of variance of logarithms of potency.

Finally, estimates of the TSH concentration for each of the patient samples were made in terms of the various calibrators, and within and between laboratory variances for the logarithms of the potencies were computed and compared.

### Results and discussion

As described in materials and methods, the study has been designed in order to analyse separately the factors contributing to assay variation, in order to determine any improvement that might be made by the use of a common assay matrix. The various levels of assay variation are summarized in figure 1.

*Analysis of intra assay monotonic 'drift'*

Analysis of coded duplicate samples (N1, N1D, N3, N3D) did not reveal any evidence of intra-assay drift or random errors, which would contribute significantly to within-laboratory and between-laboratory variability (data not shown).

*Analysis of within-laboratory, between-assay variation*

Each laboratory presented data from three separate assays. Comparisons of the three assays therefore give an estimate of within-laboratory, between-assay variation for each participant. In general, estimates for relative

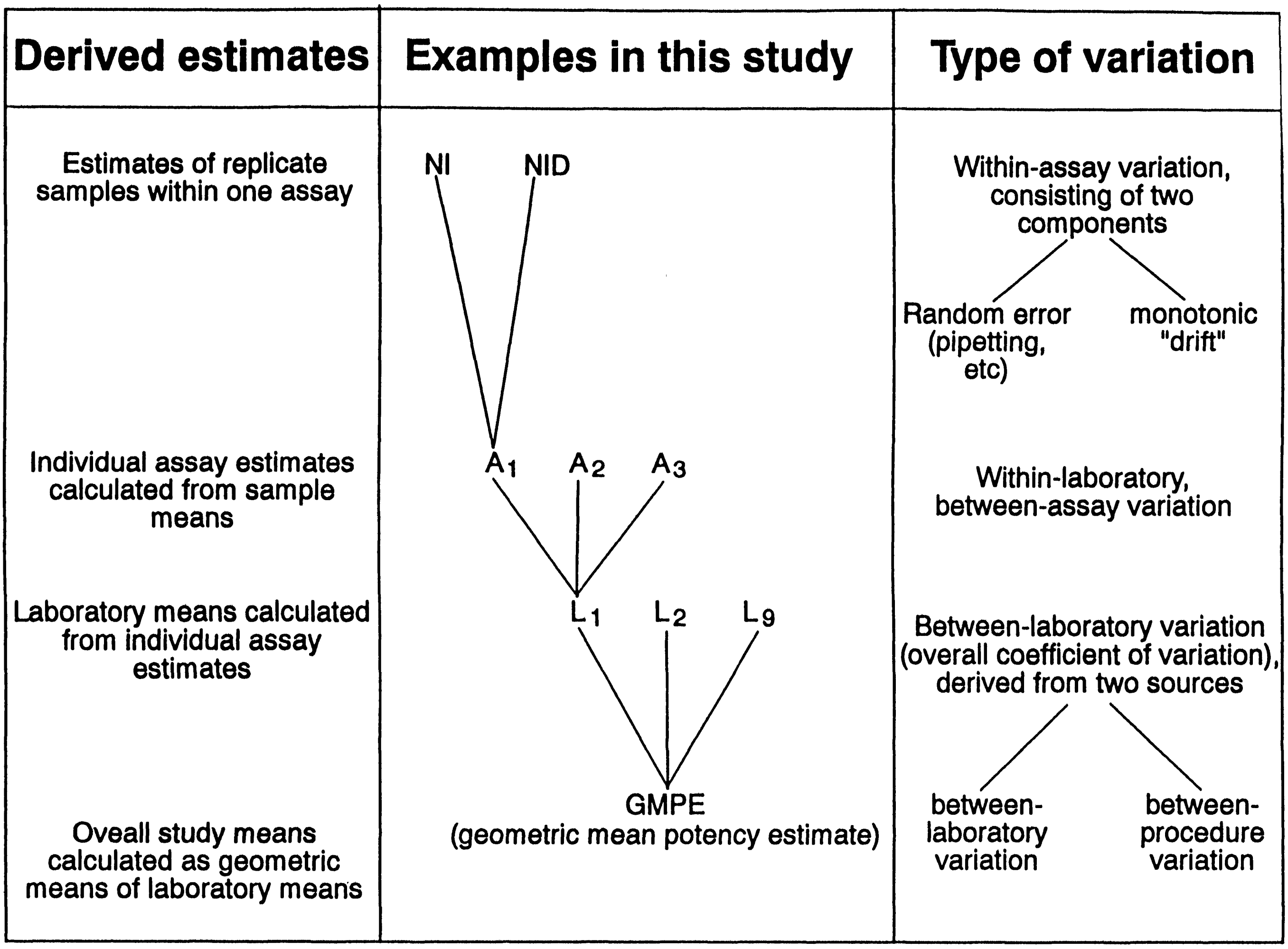

*Figure 1.*

potency of calibrator sets, 'zero' TSH calibrators and patient samples were consistent between assays, and since within-laboratory between-assay variation did not differ markedly among participants subsequent analyses of the study are presented as overall geometric means of all laboratory means, with the variance being expressed as the geometric coefficient of variation, determined from the standard deviation (SD) of the logarithms of laboratory means as (100 × (antilog(SD) −1)).

*Analysis of assay matrix effects*

Each assay contributed to this study included TSH calibrator curves prepared in nine different matrices as described in table 1. One of the matrices, that for the in-house calibrator set (KIT in tables 2 and 3 and figure 2) was individual to each participant calibrator; sets TTA, TTB, T3A, T3B and WGA used common matrices with centrally-prepared dilutions; and sets T3F, T3Y, WGX used common matrices with dilutions prepared in-house. Thus the data provided information on:

(1) The 'zero' value for each matrix.
(2) The relative potencies of calibrator sets made up in each common matrix compared to individual kit calibrators.
(3) The estimates of TSH content for patient samples using calibrator sets in each common matrix compared to those using a kit calibrator.
(4) Between-laboratory variability for estimates in terms of each calibrator, with some resolution of any improvement in that variability into components due to the use of centrally prepared dilutions, and to the use of a common assay matrix.

*'Zero' levels in low TSH matrices*

The 'zero' response for each of the nine matrices in the study was always lower than the lowest TSH dose (0·11 mU/L) in the same matrix. The different laboratories recorded different responses for the nine 'zero' doses. However, the only trend that was consistent and significant was that the 'zero' response on the WGA and WGX calibrators tended to be highest (eight out of nine laboratories) and in five out of nine laboratories was higher than the responses given by the 0·11 mU/L dose of TSH in calibrator sets in the other seven matrices. These data indicate that based on the 'zero' response criterion, matrices WGA and WGX are unsatisfactory, whilst the other seven matrices give adequately low zero responses (results not shown).

*Table 2. Overall geometric mean potency estimates and geometric coefficients of variation for various calibrators relative to selected calibrators.*

| Reference calibrator (potency = 1·0) | Geometric mean potency estimates and geometric mean CV, (%) for various calibrators relative to reference calibrator | | | | | | | |
|---|---|---|---|---|---|---|---|---|
| | T3A | T3B | T3F | T3Y | TTA | TTB | WGA | WGX |
| KIT | 1·10 | 1·11 | 1·07 | 1·06 | 1·05 | 1·02 | 1·15 | 1·11 |
| | (21) | (19) | (28) | (26) | (24) | (21) | (81) | (91) |
| TTA | 1·06 | 1·07 | 1·04 | 1·02 | — | 0·98 | 1·11 | 1·06 |
| | (10) | (13) | (15) | (15) | — | (5) | (62) | (71) |
| T3A | — | 1·01 | 0·96 | 0·95 | 0·94 | 0·92 | 1·05 | 0·99 |
| | — | (7) | (6) | (8) | (10) | (12) | (65) | (73) |
| T3Y | 1.05 | 1·07 | 1·01 | — | 0·98 | 0·97 | 1·08 | 1·04 |
| | (8) | (12) | (7) | — | (15) | (15) | (70) | (72) |
| WGX | 1·01 | 1·03 | 0·97 | 0·96 | 0·94 | 0·93 | 1·04 | — |
| | (73) | (82) | (69) | (72) | (71) | (79) | (10) | — |

Each assay contributed to the study included 9 TSH dose-response curves, for the calibrator sets described in table 1. The potency of each of these calibrator curves was estimated relative to that of a selected calibrator set. Laboratory means were pooled to give an overall geometric mean potency estimate relative to that of the reference calibrator. The geometric mean coefficient of variation (in parenthesis) is detemined from the standard deviation of the logarithms (in parenthesis) as 100× (antilog [SD]-1).

*Table 3. Overall geometric mean potency estimates (mU/ml) and geometric CV for patient samples estimates using selected calibrators.*

| Selected calibrator | Hyperthyroid samples mU/L (c.v.(%)) | | | | | Euthyroid and hypothyroid samples mU/L (c.v.(%)) | | | | | | | |
|---|---|---|---|---|---|---|---|---|---|---|---|---|---|
| | ER1 | ER2 | ER3 | ER4 | ER5 | NI | NID | N3 | N3D | N2 | N4 | O1 | O2 |
| KIT | 0·05 | 0·02 | 0·10 | 0·03 | 0·05 | 1·15 | 1·12 | 3·40 | 3·36 | 2·05 | 0·35 | 16·4 | 5·04 |
| | (80) | (131) | (66) | (21) | (33) | (25) | (28) | (21) | (21) | (29) | (48) | (38) | (23) |
| TTA | 0·05 | 0·02 | 0·10 | 0·03 | 0·05 | 1·10 | 1·08 | 3·26 | 3·24 | 1·96 | 0·34 | 15·7 | 4·85 |
| | (63) | (120) | (55) | (33) | (23) | (15) | (18) | (19) | (18) | (18) | (46) | (35) | (18) |
| T3Y | 0·05 | 0·02 | 0·11 | 0·03 | 0·05 | 1·04 | 1·00 | 3·14 | 3·12 | 1·85 | 0·33 | 15·3 | 4·70 |
| | (62) | (117) | (62) | (31) | (18) | (16) | (15) | (19) | (23) | (26) | (58) | (39) | (21) |
| WGX | 0·05 | 0·02 | 0·10 | 0·03 | 0·05 | 1·00 | 0·98 | 3·03 | 3·00 | 1·79 | 0·32 | 14·8 | 4·52 |
| | (74) | (111) | (104) | (86) | (79) | (62) | (56) | (77) | (74) | (53) | (162) | (91) | (73) |

Laboratory means for the TSH content of each of the patient samples were pooled to overall geometric mean potency estimates (mU/L). The geometric mean coefficient of variation (in parentheses) was calculated as described in table 2.

*Relative potencies or calibrator sets*

Each assay in each laboratory in the study included nine different TSH dose response curves corresponding to calibrator sets in the various matrices. By comparing one selected dose-response curve with the other eight, a set of relative potencies for the other various calibrator sets may be obtained which would reveal any effects of the assay matrix on the apparent potency in the various assays. In table 2, five selected calibrators are used as the reference. Generally, potency estimates performed in this way were close to 1·0. Only five out of 36 estimates in table 2 differed by 10% or more from the reference calibrator, with 15% being the greatest difference. These data indicate therefore, that across the whole study there was no significant effect of any matrix on the potency of the calibrator set.

It was notable, however, that the WGX or WGA calibrators, when compared with the others, gave considerably higher geometric mean coefficients of variation indicating that with these matrices some laboratories did obtain more divergent results. Comparisons of estimates in terms of T3A and T3Y did not reveal any improvement in overall between-laboratory variation as a result of the use of centrally prepared dilutions rather than in-house dilutions.

*Patient sample estimates using the various calibrators*

Overall geometric mean estimates for the 13 patient samples, obtained using KIT, TTA, T3Y or WGX calibrators are shown in table 3, and graphically in figure 2. The overall mean estimate for each sample was consistent regardless of which calibrator set was used.

*Overall coefficient of variation for patient samples using different calibrator sets*

The overall coefficients of variation for each patient sample, calculated from calibrator sets in KIT, TTA, T3Y and WGX are shown in table 3 and, graphically, in figure 1. The following conclusion may be drawn.

(1) Estimates using calibrator WGX yielded significantly higher variability than the other calibrators, due to the anomalous results that this matrix produced in some laboratories.

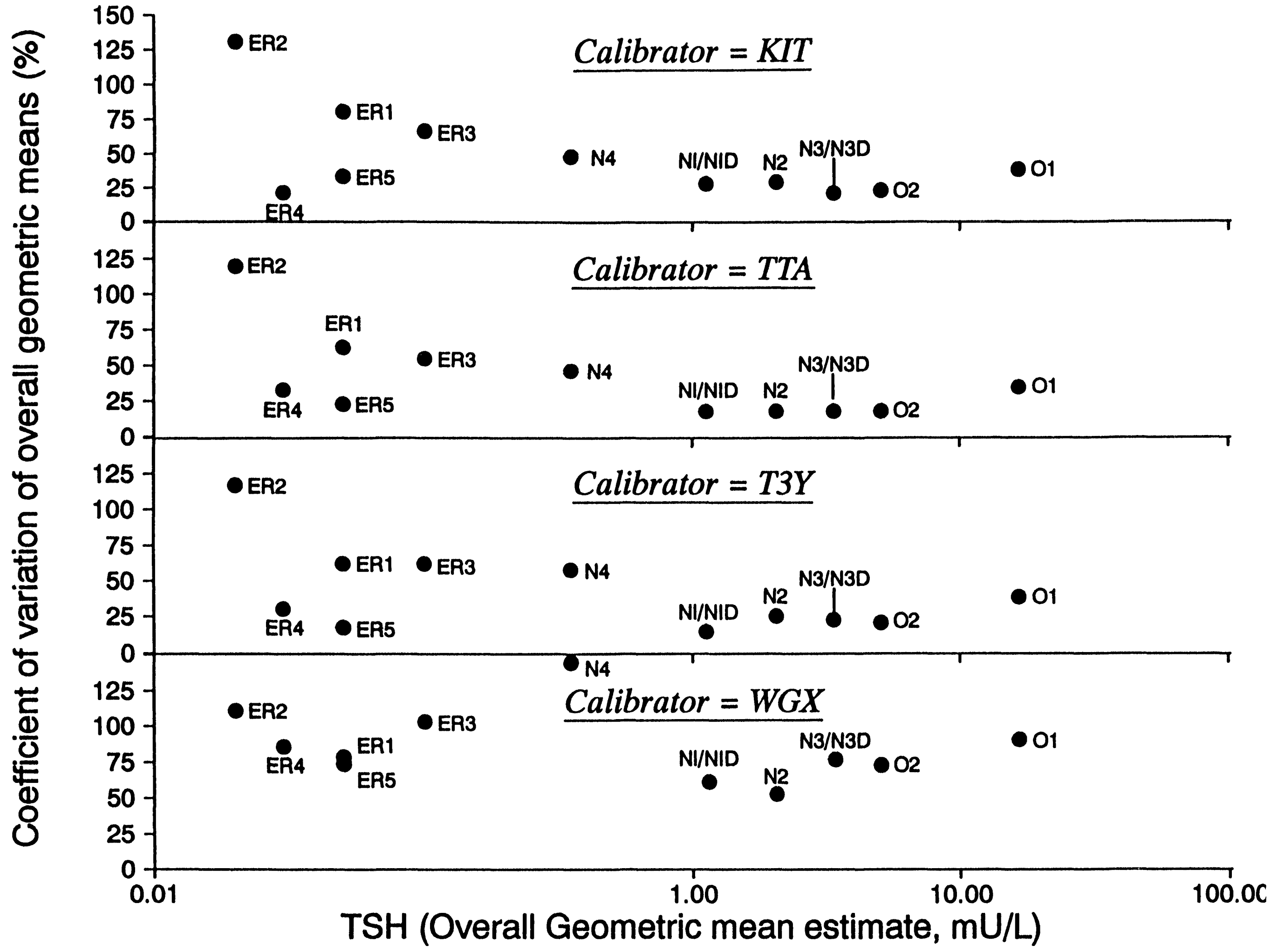

*Figure 2. Relationship between overall coefficient of variation and TSH content for patient samples. Overall geometric mean potency estimates and geometric mean coefficients of variation were calculated as described in table 3.*

(2) The use of calibrators TTA and T3X gave a marginal improvement in overall coefficient of variation for most samples when compared to KIT calibrator.

(3) With the hyperthyroid samples, the factor determining the overall variation in estimates seems to be 'sample' specific, rather than 'matrix' specific. Thus for samples ER3 (overall estimate = 0·1 mU/L) and ER1 (0·05 mU/L) the overall CVs were higher than for ER5 (0·05 mU/L) or ER4 (0·03 mU/L). These data indicate that individual assay systems may be able to discriminate qualitatively different samples, but that his discrimination would not be affected by the use of a common matrix.

### Conclusions

Two of the matrices tested (thyrotoxic serum pool or $T_3$-treated serum pool) were suitable for use as a common matrix for TSH standards based on comparisons of potency estimates, or of overall variability, and this suitability was not affected by presentation (for example lyophilized or frozen, and centrally prepared or in-house dilutions using lyophilized matrix). The third matrix (wheat-germ lectin treated serum) was not suitable in all assay systems, emphasizing that matrix effects can occur with unsuitable matrices. It remains possible that the poor performance of the wheat germ lectin treated serum was caused by its presentation in lyophilized form. This seems unlikely however since the $T_3$-suppressed serum was not similarly affected by lyophilization.

The use of a common matrix only marginally improved between-laboratory variability. This improvement is not sufficient to justify the establishment and provision of a centrally available common matix.

Variability of estimates for hyperthyroid samples may be associated with intrinsic properties of the sample, causing different reactions with different assay systems and is not improved by the use of a common matrix.

The good agreement between in-house standards and the centrally prepared calibrator sets reflects the high quality of the kits tested.

### Acknowledgements

Acknowledgements are due to the members of the WHO/IFCC working party, and, in particular, to Dr A. Swift for organizing collection of samples and matrices, to Drs

Strecker and Oellerich for testing of matrices, to Dr J. G. Loeber for provision of the wheat-germ lectin treated serum, and to the participants in the study.

### References

1. Gaines-Das, R. E. and Bristow, A. F., *Journal of Endocrinology,* **104** (1985), 367–379.

2. Gaines-Das, R. E. and Rice, L., *Computer Programs and Methods in Bioassay,* **21** (1985), 25–33.

3. Raab, G., *Applied Statistics,* **30** (1981), 32–40.

4. World Health Organization (1987), *Tech. Rep. Ser.* **745,** 11.

5. Gaines-Das, R. E. and Tydeman, M. S., *Computer Programs in Biomedicine,* **15** (1982), 13–22.

